# Fatality after deliberate ingestion of sustained-release ibuprofen: a case report

**DOI:** 10.1186/cc4850

**Published:** 2006-03-08

**Authors:** David Michael Wood, Jane Monaghan, Peter Streete, Alison Linda Jones, Paul Ivor Dargan

**Affiliations:** 1 Guy's and St Thomas' Poisons Unit, Guy's and St Thomas' NHS Foundation Trust, London, UK

## Abstract

**Introduction:**

Ibuprofen is a nonsteroidal anti-inflammatory drug available over the counter and on prescription for the management of pain and inflammation. Severe toxicity is rare following deliberate self-poisoning with ibuprofen, and patients are usually either asymptomatic or develop only mild gastrointestinal toxicity. Although there have been nine other reported fatalities, co-existent factors have probably contributed to all of these deaths. We report here a fatality from isolated toxicity following self-poisoning with sustained-release ibuprofen.

**Case report:**

A 26-year-old female presented after deliberate ingestion of up to 105 g sustained-release ibuprofen, with a reduced level of consciousness, severe metabolic acidosis and haemodynamic compromise. Despite intensive supportive management, gut decontamination with multidose activated charcoal and correction of the metabolic acidosis with sodium bicarbonate and haemofiltration, the patient did not survive. The ibuprofen concentration *ante mortem *on presentation in peripheral blood was 760 mg/l and the concentrations *post mortem *were 518 mg/l in peripheral blood, 74 mg/kg in liver extract and 116 mg/l in the gastric contents.

**Discussion:**

Most patients with ibuprofen poisoning are either asymptomatic or have mild gastrointestinal symptoms; severe poisoning with ibuprofen is rare. We report the first death related to isolated sustained-release ibuprofen poisoning.

## Introduction

Ibuprofen is a nonsteroidal anti-inflammatory drug (NSAID) commonly used as an analgesic, as an anti-inflammatory agent and as an anti-pyretic agent [1,2]. The predominant pharmacological action of ibuprofen is to inhibit the activity of cyclooxygenase, an enzyme crucial for the synthesis of prostaglandins. The subsequent inhibition of prostaglandin production leads to a reduction in inflammation, temperature and pain, both centrally and peripherally. Ibuprofen is manufactured and marketed as a 'normal' release preparation at a dose of 400 mg three times a day or a sustained-release preparation at a dose of 800–1600 mg once a day. In the United Kingdom the 'normal'-release preparation is available on general sales licence, pharmacy and prescription, but the sustained-release preparation is available only as a 'prescription only medication'.

There have been only nine previously reported fatalities following ibuprofen intoxication, although in eight of these cases other co-existent factors have probably contributed to death [3-11]. We report here the first case report of a fatality following isolated ingestion of sustained-release ibuprofen that did not respond to maximal supportive care with *ante mortem *and *post mortem *ibuprofen concentrations.

## Case report

A 26-year-old woman with no significant past medical history presented after ingestion of up to 132 tablets of 800 mg sustained-release ibuprofen, equivalent to approximately 105 g. This estimate of the amount ingested was based on empty ibuprofen packets found near her. The patient was bought into the Emergency Department having been found collapsed and unconscious at home by her family, who had last seen her well approximately five hours previously. There was no history of vomiting, gastrointestinal haemorrhage or seizures prior to presentation at hospital. Her initial Glasgow Coma Scale was 3/15 and the patient was therefore intubated and ventilated to provide a protected airway. On presentation she was haemodynamically compromised with a systolic blood pressure of 80 mmHg. The patient's initial electrocardiogram showed sinus rhythm, normal QRS duration and normal QT duration, but widespread myocardial ischaemia was noted. Initial biochemistry blood test results were sodium 132 mmol/l, potassium 4.7 mmol/l, urea 4.8 mmol/l, creatinine 159 μmol/l and venous blood glucose 4.7 mmol/l. Paracetamol and salicylate concentrations were not detected on her admission blood samples. Arterial blood gases showed a severe metabolic acidosis with pH 6.99, base excess of -21 and lactate of 17 mmol/l. The patient was commenced on epinephrine and norepinephrine for inotropic support in view of the significant hypotension, and the Guy's and St Thomas' Poisons Unit was contacted for further advice on management.

Since this was potentially a life-threatening ingestion of a sustained-release preparation of ibuprofen, it was recommended that multidose activated charcoal (50 g activated charcoal every 3–4 hours) should be given via a nasogastric tube to try and reduce further absorption of ibuprofen from the gastrointestinal tract. The patient's severe metabolic acidosis should be corrected with repeated doses of intravenous boluses of 8.4% sodium bicarbonate, and haemofiltration with a bicarbonate buffer if the metabolic acidosis did not respond to intravenous sodium bicarbonate. Other potential common drug and toxicological causes of a high anion gap lactic acidosis are summarised in Table 1. It should be ensured that the patient is adequately filled with intravenous fluid to sustain blood pressure prior to the commencement of any additional inotropic support.

Despite fluid resuscitation and maximal infusion doses of epinephrine and norepinephrine, the patient remained hypotensive with a systolic blood pressure of 80 mmHg. Additionally her metabolic acidosis remained resistant to intravenous sodium bicarbonate and haemofiltration with a bicarbonate buffer, with only minor improvement to pH 7.00. Her clinical condition continued to deteriorate and approximately five hours post-presentation to the Emergency Department the patient suffered a ventricular tachycardia/ventricular fibrillation cardiac arrest, which did not respond to standard Advanced Life Support protocol cardiopulmonary resuscitation.

## Results

### Serum toxicology screening

Samples of *ante mortem *serum were obtained following admission and were analysed for ibuprofen by the Medical Toxicology Laboratory in London. *Post mortem *samples of peripheral whole blood, urine, gastric contents and liver extract were analysed at the local toxicology laboratory for ibuprofen and other drugs. Ibuprofen concentrations were measured by high-pressure liquid chromatography with ultraviolet detection. *Ante mortem *serum ibuprofen concentrations were 760 mg/l on presentation, rising to a peak concentration of 1,050 mg/l 90 minutes after presentation. *Post mortem *ibuprofen concentrations were 518 mg/l, 264 mg/l, 116 mg/l and 74 mg/kg in the peripheral whole blood, urine, gastric contents and liver extract, respectively. No other drugs were detected in a broad toxicology screen; analysis of the *ante mortem *and *post mortem *serum samples only detected atracurium and lignocaine given following admission to the hospital.

### Post mortem

The cause of death was probably directly related to the ibuprofen overdose, since there was no evidence of another cause of death at the *post mortem *examination. Of particular note there was no evidence of cerebral oedema, no underlying artherosclerotic disease of the coronary arteries and no evidence of previous myocardial infarction. Although there was altered blood in the gastric fluid, there was no evidence of oesophageal or gastric erosions.

## Discussion

Severe poisoning and death following poisoning with ibuprofen is extremely uncommon. Most cases are either asymptomatic or experience mild gastrointestinal symptoms only [4,5]. In the case presented here the patient presented after ingestion of up to 105 g sustained-release ibuprofen with a reduced Glasgow Coma Scale, a severe metabolic acidosis and significant haemodynamic compromise. Despite meticulous supportive care initially in the Emergency Department and subsequently in the intensive care unit, attempted correction of her metabolic acidosis and the use of multidose activated charcoal to reduce further ibuprofen absorption from the gastrointestinal tract, the patient did not survive. This is the first reported case of fatality following ingestion of sustained-release ibuprofen and the first fatality following isolated ibuprofen toxicity.

Ibuprofen is a NSAID commonly used as an analgesic, as an anti-pyretic agent and as an anti-inflammatory agent [1,2]. The predominant pharmacological effect of ibuprofen, similar to other NSAIDs, is to inhibit the activity of cyclooxygenase (both COX-1 and COX-2), leading to an inhibition of prostaglandin synthesis. Following a therapeutic dose of 400 mg, the serum ibuprofen concentration is approximately 28 mg/l (range 17–36 mg/l) [12]. Clinical features of toxicity of ibuprofen and other NSAIDs are predictable and occur due to an inhibition of cyclooxygenase activity.

The American Academy of Clinical Toxicology and European Association of Poisons Centres and Clinical Toxicologists have published a position statement on the use of multidose activated charcoal [13]. This position statement, however concentrated on the evidence base for the increased elimination of drugs undergoing enterohepatic/enteric circulation, rather than reducing the absorption of sustained-release or modified-release preparations.

In the case reported here a sustained-release preparation of ibuprofen was ingested, and therefore multidose activated charcoal was recommended to try and reduce further absorption of ibuprofen. The *post mortem *gastric content ibuprofen concentration was 116 mg/l, suggesting a significant amount of ibuprofen had still not been absorbed more than five hours post-presentation to the Emergency Department. Another patient who was found dead who had recently been prescribed an 800 mg preparation of ibuprofen, presumed to be a sustained-release preparation, had a *post mortem *total ibuprofen concentration of 131 mg in the gastric contents [8]. Both our case and the other presumed sustained-release case would support the use of multidose activated charcoal in the management of patients who have ingested a sustained-release preparation of ibuprofen in any subsequent cases.

The toxicity of ibuprofen following self-poisoning has been reported in five large case series [3-5,10,14]. Between 80% and 90% of the patients in these case series were either asymptomatic or had mild gastrointestinal symptoms, such as nausea, vomiting and diarrhoea, following ibuprofen intoxication [3-5]. Several case series have demonstrated that, in patients with a history of ingestion of less than approximately 100 mg/kg ibuprofen, symptoms did not occur [4,5,15] and that symptoms of ibuprofen toxicity following ingestion of a standard release preparation usually occur within four hours of ingestion [4,5].

Severe toxicity is uncommon following ibuprofen self-poisoning, and in general less than 10% of patients develop 'life-threatening' symptoms such as coma, seizures, respiratory arrest, hypotension or anuric renal failure [3-5,10]. Life-threatening features of ibuprofen toxicity have been shown only to occur in patients who have ingested greater than 400 mg/kg ibuprofen [15]. Histories in patients presenting with an overdose have been shown to be unreliable [16], however, so to try and predict those patients who are at risk of severe ibuprofen-induced toxicity, a nomogram based on the time since ingestion and the serum ibuprofen concentration, similar to that used for paracetamol (acetaminophen), has been developed [4]. Subsequent studies have shown conflicting results as to whether this nomogram is accurate [5] or inaccurate [10] at predicting those at risk of severe toxicity. Since ibuprofen concentrations are not routinely available in most emergency departments or hospitals, there are concerns about the accuracy of the nomogram, the toxic effects of ibuprofen are predictable and (unlike paracetamol poisoning) there is no effective antidote, we would not recommend use of the ibuprofen nomogram in routine clinical practice.

Management of patients presenting following deliberate self-poisoning with ibuprofen consists of gut decontamination with activated charcoal, if they present within one hour of a potentially toxic overdose, and generalised supportive care [17,18]. As already discussed, multidose activated charcoal may be appropriate in patients who have ingested a potentially toxic amount of a sustained-release preparation. Other more severe features of ibuprofen toxicity should be managed appropriately. Ibuprofen-induced seizures that are nonself-limiting should initially be managed with intravenous diazepam (0.1–0.2 mg/kg). Significant metabolic acidosis (pH < 7.0) that does not respond to adequate intravenous fluid resuscitation, and maintenance of the blood pressure, with intropic support if appropriate, should be corrected with intravenous 50–100 ml boluses of 8.4% sodium bicarbonate. For resistant metabolic acidosis that is not responding, then haemofiltration with a nonlactate bicarbonate buffer may be beneficial. Although ibuprofen has a relatively low volume of distribution (0.1 l/kg), its high protein binding to albumin (99%) limits removal by extracorporeal treatments such as haemodialysis or haemofiltration [19].

Previous studies have demonstrated no accumulation of ibuprofen in patients with renal impairment [20] and, in functionally anephric patients undergoing renal replacement therapy with haemodialysis, no accumulation of ibuprofen was seen and there was no detectable ibuprofen in the dialysate, indicating that the ibuprofen was eliminated through metabolism [21]. This provides further support that extracorporeal treatments will probably not be beneficial in increasing the clearance of ibuprofen in overdose, and there have been no previous reported cases of their attempted use in patients with ibuprofen toxicity. There have been no published studies on the routine prophylactic use of H_2 _histamine receptor antagonists or proton pump inhibitors in trying to reduce the risk of ibuprofen or other NSAID-related gastrointestinal toxicity. Our current practice in patients with significant epigastric pain/tenderness after ibuprofen poisoning is to treat them with 1 week of a proton pump inhibitor such as lansoprazole 30 mg once daily.

There have been nine reported cases of fatality following ibuprofen self-poisoning in the literature to date, although other factors probably contributed to death in eight of these cases [3-11]. The co-ingestion of other drugs at the time of the overdose, such as aspirin, paracetamol, theophylline and cyclobenzaprine, contributed to death in four cases [3,6,7,9]. Aspiration pneumonia that developed as a complication of ibuprofen-induced apnoeic episodes [4] and septic shock, thought to be unrelated to ibuprofen toxicity [10], contributed to two deaths. Refusal of treatment of ibuprofen-induced oliguric renal failure and sepsis, felt by the authors to be survivable, significantly contributed to one death [5]. The circumstances surrounding one death are unclear as the patient was found dead near their home [8]. There are limited details of and no confirmatory ibuprofen concentrations for the final death, which has been reported in abstract form only [11].

Ibuprofen concentrations have been measured in four of the previous fatalities [6,8-10]. One of the previously reported fatalities had an *ante mortem *ibuprofen concentration of 72 mg/l; although few details of the case were given, the authors concluded that the cause of death was septic shock and respiratory failure unrelated to the ibuprofen intoxication [10]. Peripheral blood *post mortem *ibuprofen concentrations of 81 mg/l, 130 mg/l and 348 mg/l have been reported in a 48-year-old male [6], a 19-year-old male [9] and a 26-year-old male [8], respectively.

Additionally, *post mortem *ibuprofen concentrations of 942 mg/kg [8] and 238 mg/kg [6] were reported in liver extract in two cases. In the case reported here, the *post mortem *ibuprofen concentrations were 518 mg/l in peripheral blood and 74 mg/kg in liver extract. The main differences between our reported case and the other two cases with previous reported *post mortem *ibuprofen concentrations is that our case had higher peripheral blood and lower liver extract concentrations. Since the exact timing of ingestion was not known in our case and was not reported in the other two cases, the differences in peripheral blood and liver extract ibuprofen concentrations may be due to differences in distribution and metabolism. It is therefore probable, given the *post mortem *ibuprofen concentrations in our reported case, that our patient died sooner after ingestion than the other two reported cases, as peripheral blood concentrations had not had sufficient time to fall and the liver had not started to metabolise as much ibuprofen. The other unknown factor in all of these cases is the impact of impaired haemodynamics, renal dysfunction and metabolic acidosis on ibuprofen kinetics.

## Conclusion

We have described the case of a fatality following severe poisoning with sustained-release ibuprofen. The patient presented with a reduced Glasgow Coma Scale, severe metabolic acidosis and haemodynamic compromise that did not respond to meticulous supportive care, to treatment with sodium bicarbonate, to haemofiltration and to inotropic support. There were no other toxicological or medical causes for the patient's clinical presentation. Multidose activated charcoal was utilised in this patient due to the ingestion of a sustained-release preparation, and its use was supported by elevated ibuprofen concentrations in the gastric contents following death.

## Key messages

• 	Ibuprofen is a NSAID used as an analgesic, as an anti-pyretic agent and as an anti-inflammatory agent.

• 	Most patients with ibuprofen overdoses are usually asymptomatic or have mild gastrointestinal symptoms.

• 	Symptoms are unlikely if less than 100 mg/kg ibuprofen has been ingested.

• 	Symptoms of severe ibuprofen toxicity, including metabolic acidosis, seizures, renal impairment and cardiovascular collapse, occur after >400 mg/kg has been ingested.

• 	Patients require meticulous supportive care and management of ibuprofen-induced complications.

## Abbreviations

NSAID = nonsteroidal anti-inflammatory drug.

## Competing interests

The authors declare that they have no competing interests.

## Authors' contributions

DMW, PID, ALJ and JM provided toxicology advice on the management of the patient. PS analysed the ibuprofen samples. DMW and PID drafted the first draft of this manuscript. All authors contributed to the final draft of the manuscript.

## Figures and Tables

**Table 1:** Common toxicological causes of a high anion gap lactate acidosis (adapted from 22)

Biguanides (for example, metformin)
Cyanide
Iron
Salicylates
Theophylline
Type B lactic acidosis (for example, from hypotension related to any significant poisoning)
